# Application of Thin-Film Microextraction to Analyze Volatile Metabolites in A549 Cancer Cells

**DOI:** 10.3390/metabo11100704

**Published:** 2021-10-14

**Authors:** Wojciech Filipiak, Karol Jaroch, Paulina Szeliska, Karolina Żuchowska, Barbara Bojko

**Affiliations:** Department of Pharmacodynamics and Molecular Pharmacology, Faculty of Pharmacy, Collegium Medicum in Bydgoszcz, Nicolaus Copernicus University in Toruń, Dr. Jurasza 2 Str., 85-089 Bydgoszcz, Poland; karol.jaroch@cm.umk.pl (K.J.); p.modrakowska@cm.umk.pl (P.S.); karolina.zuchowska@doktorant.umk.pl (K.Ż.); bbojko@cm.umk.pl (B.B.)

**Keywords:** TFME, SPME, volatile metabolites, VOCs, A549, cancer cells, in vitro models, cellular metabolites, extraction techniques, GC-MS

## Abstract

Volatile organic compounds (VOCs) have been proposed in the last two decades as biomarkers for disease detection and therapeutic monitoring. Model in vitro experiments with established cell lines are fundamental to clarify whether given VOCs originate from normal human cells or pathogens, including transformed cancer cells. Due to the trace concentrations of target metabolites, adsorptive enrichment is needed before gas chromatography-mass spectrometry (GC-MS) analysis, with solid-phase microextraction (SPME) being perfectly suited for this purpose. Here, a modification of SPME, the thin-film microextraction (TFME) technique, is proposed for analysis of cellular VOCs, which utilizes a planar mesh coated with stationary phase to increase the extraction phase volume and active surface area. In this study, four different adsorbents were compared: carboxen, divinylbenzene, hydrophobic−lipophilic balanced and polydimethylsiloxane. Amongst them, HLB sheets using poly(divinylbenzene-co-N-vinyl-pyrrolidone) skeleton structure proved to be the most versatile, enabling the most sensitive analysis of VOCs with a broad polarity and volatility. For HLB, sampling type (internal static headspace, external bi-directional headspace), extraction temperature and extraction time were also examined. An established method was successfully applied to analyze metabolites produced by A549 cells revealing five volatiles at significantly higher (additionally benzaldehyde at lower) levels in cell culture medium compared to the cell-free reference medium headspace.

## 1. Introduction

Non-small cell lung cancer (NSCLC) is still one of the most common cancer diseases, with a five year survival rate varying from 92 to 0% [1] depending on the cancer stage at initial diagnosis. Due to the low specificity of symptoms (e.g., cough) NSCLC is often diagnosed at a late stage of the disease, with approximately 38% of patients being diagnosed at stage IV [2]. Thus, an early, easy to handle, and relatively cheap screening test is desired to improve current methods for cancer detection. Nowadays, volatile organic compounds (VOCs) are receiving increasing interest as potential biomarkers related to lung cancer [3], but their trace concentrations require a preconcentration step prior to offline instrumental analysis. In this regard, solid-phase microextraction (SPME) is a widely used preparation technique that relies on obtaining equilibrium between analyte concentration in a sample and the amount extracted by the device [4]. Various formats of SPME devices are commonly applied for analysis of liquid samples in clinical and pharmacological research, whereas for preconcentration of volatile metabolites from gaseous samples, small fibers made of fused silica (or nitinol) coated with a chosen sorptive phase are the gold standard [5]. SPME fibers have been proven to extract VOCs from diverse human body fluids including exhaled breath samples, tissues from stomach cancer patients [6] and from lung cancer patients [7,8]. For in vitro models of lung cancer, the A549 adenocarcinoma cell line is often employed, and SPME fibers have been successfully applied to VOCs extraction from a cell culture headspace [9], even during their growth phase [10]. A pharmacometabolomic study on cisplatin influence on volatilome of A549 cells has been investigated utilizing SPME fibers, with particular VOCs shown to be related to cell apoptosis [11].

Due to limited extraction efficiency resulting in relatively low sensitivity of fiber-based SPME devices, thin film microextraction (TFME) has been developed. TFME can have diverse geometry, for instance, a metal blade covered with solid sorbent immersed in polyacrylonitrile glue, which is commonly used in Liquid-Phase Microextraction including Direct Immersion Single Drop Microextraction (DI-SDME) [12]. Another option is a mesh-like geometry, where the polymer particles of the extraction phase are immersed in biocompatible glue made of Poly(dimethylsiloxane) (PDMS) and a prepared slurry is sprayed to form a thin layer on carbon mesh. This structure is highly resistant to temperature, allowing thermal desorption of extracted analytes, making this variant suitable for analysis of volatile compounds by means of gas chromatography [13]. Different chemical compositions of the extraction phase may be used to adjust sample preparation protocol, either broadening the range of analytes for untargeted analysis or narrowing it to the target group, considering the analytes polarity, volatility and its concentration in a sample, as well as the complexity of the sample matrix [13]. In this regard, strong adsorbents such as Carboxen are recommended for extraction of trace levels of highly volatile analytes of small molecular weight (<150). However, larger molecules, especially those containing functional groups responsible for π-π interactions (e.g., conjugated double bonds), bind to the microporous carbon surface too strongly and may not allow sufficient desorption [14]. For this reason, mixed-phases are used in the SPME technique, where fibers composed of a layer of divinylbenzene (DVB) suspended in polydimethylsiloxane (PDMS) and coated with a layer of Carboxen (CAR) are the most common choice for extraction of volatiles with a wider molecular weight range. However, the limitation of this approach is a reduced sample capacity due to the thinner coating of each sorbent, hindering the analysis of trace-VOCs in a complex matrix (containing far more abundant interfering substances).

In the current work, we present, for the first time, an application of TFME to the analysis of volatile metabolites in the headspace of Adenocarcinoma cell cultures. Four different sorbents were compared, including CAR, DVB, PDMS and HLB, and optimal extraction conditions were selected for the chosen material. The A549 in vitro model was used to demonstrate the usefulness of the TFME technique in the investigation of volatile metabolites originating from cancer cells.

## 2. Results and Discussion

In the in vitro studies focused on the A549 volatilome, diverse sampling techniques were used, inter alia, an exhaustive technique with a packed sorption tube monolith monotrap, but in most cases VOCs were extracted on SPME fibers coated either with carboxen-divinylbenzene [8,10,15], or a carboxen [16] phase. The in vitro SPME sampling was performed by placing aqueous medium in a gas tight vial followed by preincubation and exposure of a fiber assembly to the headspace [15]. In another protocol, we exposed the SPME fiber directly to the A549 cell headspace inside cultivation bottles (flasks) stored in an incubator [9,10,16]. In ref [9,16] cultivation flasks were separated from the incubator atmosphere by turning caps to the gas tight position 1 h before sampling. Although such an approach allows for a “cleaner” background (lesser exogenous VOCs), lack of CO_2_ may have an impact on cell growth. A novel approach utilizing a silica monolith monotrap attached with a syringe to the cultivation flask with a modified gas-tight cap was recently proposed by Furuhashi et al. [17]. In this protocol, sample flow through the monotrap was generated by bidirectional movement of the syringe plunger for the entire extraction time. Another sampling method applied in the study of A549-derived VOCs used TD sorption tubes, where a continuous flow of purified synthetic air enriched with 5% CO2 was used as a carrier gas to draw headspace sample through a solid adsorbent [18]. In turn, ex vivo tissue sampling was performed by placing resected lung tissue in a gas-tight vial followed by exposure of the SPME fiber for static headspace sampling [8], or by purging the vial content with synthetic air with an outlet connected to a sorbent-packed tube [19]. In this work, the protocol for Thin-Film Microextraction is optimized and presented for the first time to analyze emission of volatile metabolites from A549 cancer cells.

Altogether 68 VOCs were detected in all headspace samples from A549 cells using the GC method described here. Out of these, 40 reference substances were available for us to unambiguously confirm identification of metabolites. Hence, to ensure high reliability of the data, we focused only on those 40 VOCs in optimization of the TFME sampling procedure and further statistical evaluation of results gathered for A549 cells. Compounds for which a statistically significant difference was observed between the A549 cell culture medium and the cell-free reference medium were further calibrated and Detection Limits with Quantitation Limits were determined according to the European Medicines Agency guidelines. Metabolites which did not fulfill the EMA recommendations were excluded from further consideration.

### 2.1. Optimization of the TFME Extraction Procedure

**Figure 1:**
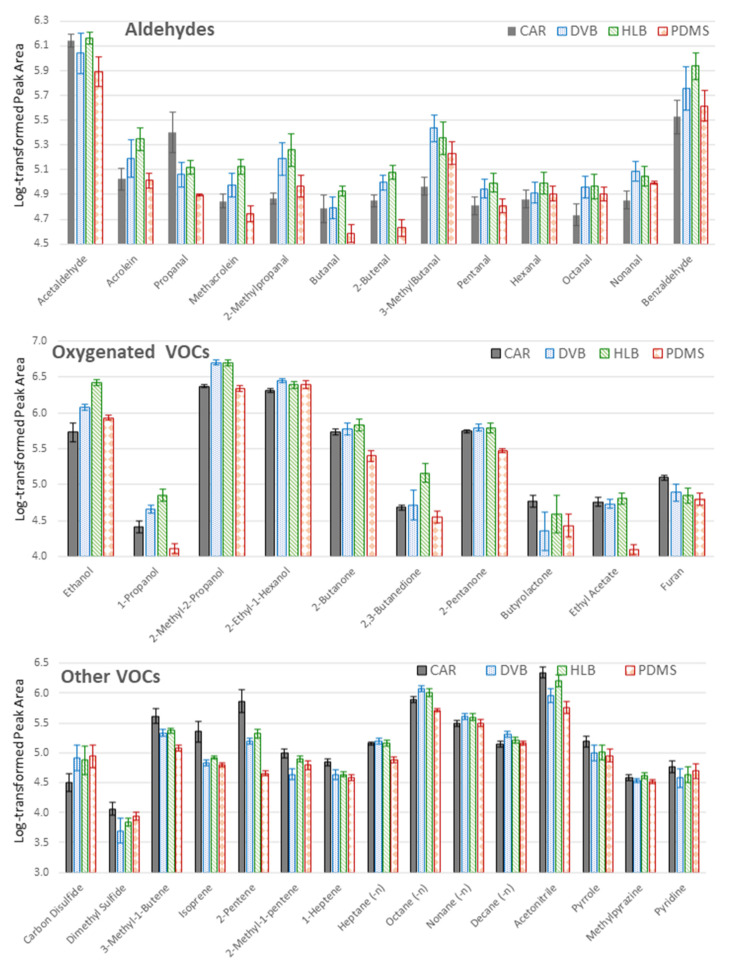
Sorbent optimization of volatile organic compounds (VOCs). The *y*-axis represents log-transformed peak area values. Mean values are given with standard deviation for four replicates (*n* = 4) for each sorbent: Carboxen (CAR, grey boxes), divinylbenzene (DVB, blue boxes), hydrophobic−lipophilic balanced (HLB, green boxes), polydimethylsiloxane (PDMS, red boxes).

The first stage of optimization of the TFME procedure showed that all unambiguously identified VOCs (*n* = 40) were detected, regardless of sorbent type used (Figure 1). For highly volatile molecules, *inter alia*, acetaldehyde, propanal, acetonitrile, isoprene, dimethyl sulfide, 3-methyl-1-butene, pyrrole, very good results were obtained for Carboxen sheets. Carboxen had the largest active surface area (>1000 m^2^/g) with microporous structure, which resulted in the highest adsorption strength amongst all sorbents investigated here. However, for alcohols observed in this study, i.e., ethanol, 1-propanol, 2-methyl-2-propanol, and 2-ethyl-1-hexanol, considerably better extraction efficiency was observed for DVB and HLB sheets compared to Carboxen. This may be related to the displacement of those polar molecules containing hydroxyl group with water molecules (the RPMI medium is an aqueous solution) which have higher adsorption affinity to Carboxen than the mentioned VOCs. Additionally, Carboxen may appear to be too strong for larger molecules, especially those containing functional groups responsible for π-π interactions. In this case, the temperature applied may be insufficient for complete desorption on the one hand, but further increase of temperature may damage the PDMS support under Carboxen on TFME sheet on the other hand. This is in line with the results gathered, whereby heptane, octane, nonane and decane were most efficiently extracted with DVB meshes. Divinylbenzene is nonpolar sorbent in which nonspecific Van der Waals forces and π-π interactions play a major role. However, the polar functional groups present in the target molecule weaken those interactions. Basically, the more electronegative a functional group, the weaker are the π-π interactions between the sorbent and analyte molecule. For this reason, the N-vinyl-pyrrolidone groups are built into the divinylbenzene skeleton yielding more polar hydrophilic-lipophilic balanced (HLB) sheets. This is supported by an observation in this study, where oxygenated VOCs, including alcohols, ketones and aldehydes, were extracted with the highest efficiency on HLB (Figure 1). In turn, PDMS membranes resulted in the worst metabolite coverage, with only carbon disulfide being extracted with higher efficiency compared to other sorbent types.

**Figure 2:**
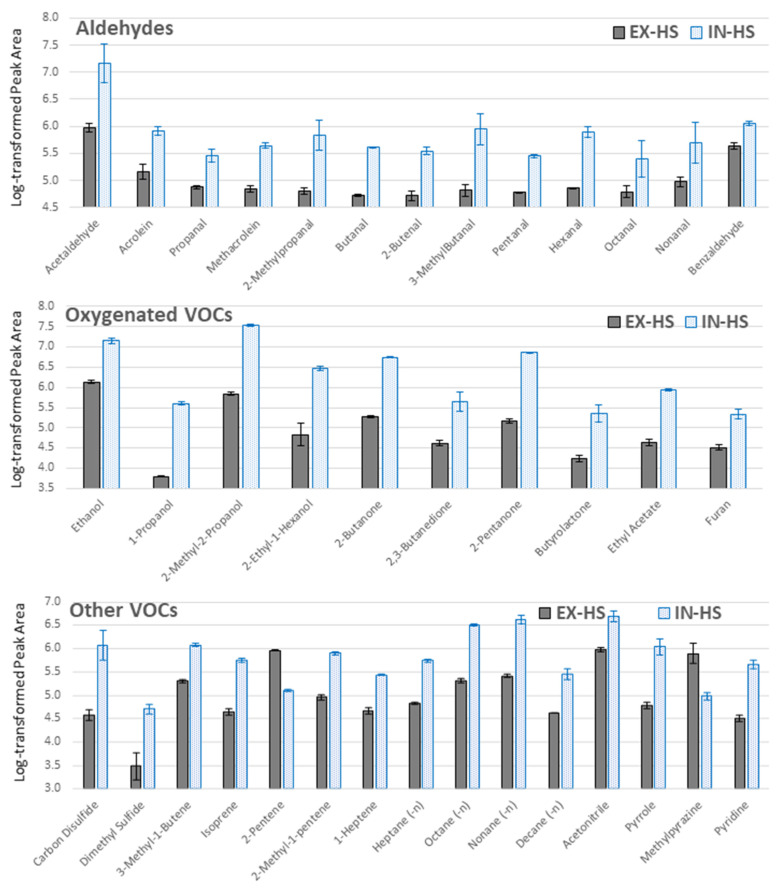
Optimization of sampling protocol for VOCs extraction from a pooled medium headspace. The *y*-axis represents log-transformed peak area values, where means are given with a respective standard deviation for three replicates (*n* = 3) for each sampling mode: external headspace (EX-HS, grey boxes) and internal headspace (IN-HS, blue boxes).

Special care should be taken when analyzing biological samples, particularly infectious material such as bacteria or infected human fluids. For this reason, the external sampling mode (EX-HS) was investigated in which the TFME sheet was separated from the sample by placing it inside a TD tube (fixed with a tiny portion of glass wool). Although the headspace sample was drawn through the TFME sheet by multiple bidirectional movements of the plunger of the 2 mL syringe, after which the sorbent was still exposed to the sample via 1/4″ Teflon tube (over 30 min), the extraction efficiency in this protocol was worse compared to the internal HS mode (IN-HS) for all VOCs detected, except 2-pentene and methylpyrazine (Figure 2). It should be noted that in the IN-HS mode, the initially generated headspace was broken for a short time (3–5 s) by opening a sample vial to replace an original cap with one containing a sorbent sheet. Nonetheless, quick achievement of a new equilibrium in a relatively small, closed system (5 mL liquid medium in 20 mL vial) and fast extraction kinetics on TFME mesh, along with no restriction to VOCs diffusion from the headspace to the sorbent (in contrast to deteriorated transfer through glass wool into a TD tube), resulted in higher extraction rates for IN-HS compared to EX-HS.

Although an ambient extraction temperature of ~20 °C is very convenient, it has important drawbacks, namely it is not optimal for cultured cell lines and microorganisms, and it may be unstable within an intra and interday period. Therefore, a temperature of 37 °C for TFME extraction was also studied using a sampling protocol elucidated from previous measurements, i.e., HLB material and IN-HS mode.

**Figure 3:**
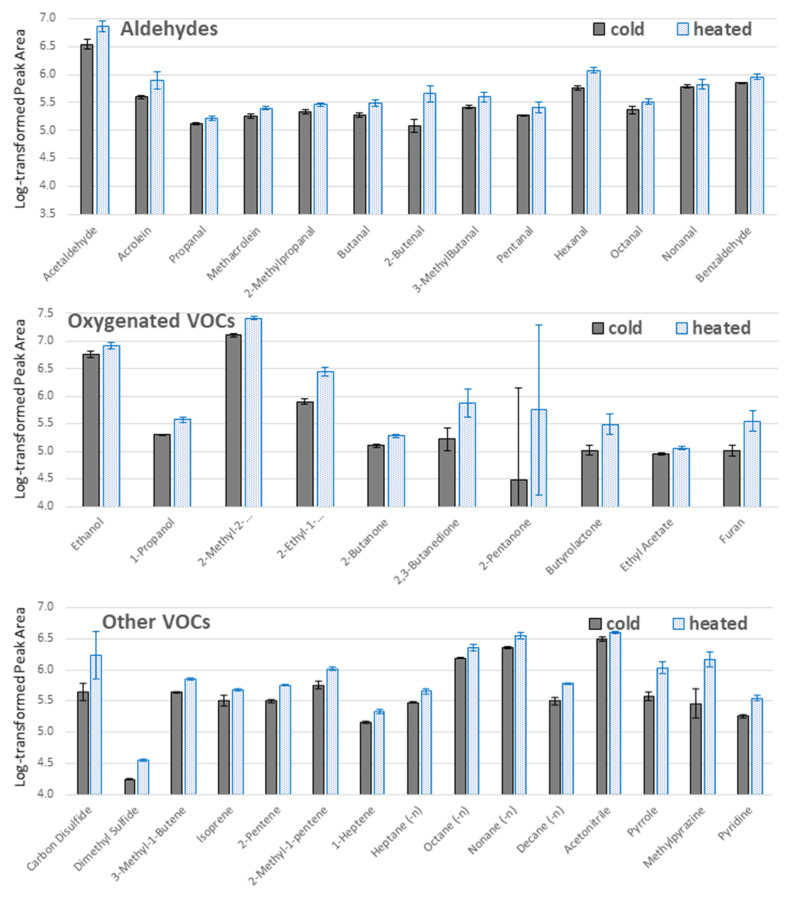
Influence of temperature on VOCs extraction from a pooled medium headspace on HLB sheets according to IN-HS sampling protocol. The *y*-axis represents log-transformed peak area values, where means are given with a respective standard deviation for three replicates (*n* = 3) for each temperature: ambient of ~20 °C (“cold”, grey boxes) and elevated to 37 °C (“heated”, blue boxes).

According to the obtained results, an elevated temperature improved extraction efficiency for all VOCs detected (Figure 3). This is a consequence of enhanced vaporization of volatile compounds from the aqueous medium, hence their faster distribution within the headspace gas and better penetration of the sorbent solid phase. Simultaneously, the temperature of 37 °C seems to be sufficiently low to avoid undesired depletion in the exothermic adsorption process.

**Figure 4:**
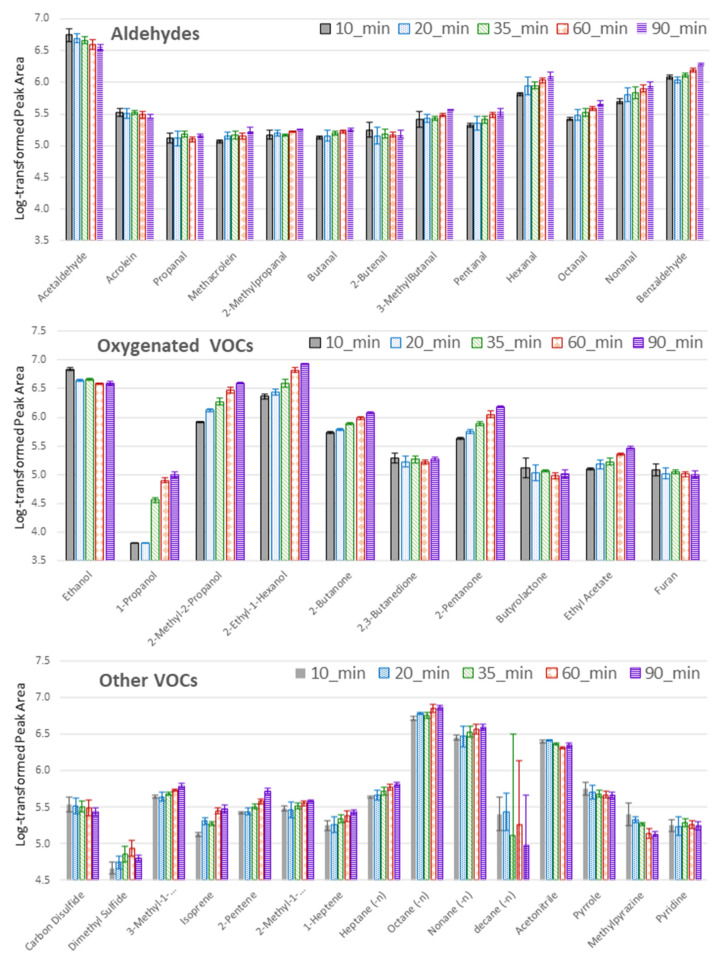
Optimization of extraction time. The *y*-axis represents log-transformed peak area values, where means are given with a respective standard deviation for three replicates (*n* = 3) for each extraction time: 10min (grey boxes), 20min (blue boxes), 35min (green boxes), 60 min (red boxes), 90 min (magenta boxes).

In the search for improved sensitivity, so much needed in ultra-trace analysis, the extraction phase volume is often enlarged. Despite the obvious benefit of increased capacity for the analyte, this approach results in a prolonged equilibration time and hinders desorption efficiency. Therefore, in TFME, the geometry of the device is designed to ensure an effectively high sorbent area-to-volume ratio by deposition of an extraction phase on the flat planar mesh. Grandy et al. [13] demonstrated that this process was crucial to decrease the background from the TFME device itself, whereby high-density PDMS prepolymer was used as a thermally stable glue to support the extraction phase on a carbon (instead of fibreglass) mesh yielding minimal bleeding of siloxanes. Given the importance of the extraction time in SPME sampling, the following durations were tested: 10, 20, 35, 60 and 90 min. According to the results gathered, the higher the molecular weight (and lower volatility), the longer time needed (Figure 4). This is evident for aldehydes and particularly for alcohols, where the initially weak influence of extraction time on amounts of adsorbed small molecules within those groups increased for heavier compounds. A similar relationship was maintained over the full range of hydrocarbons investigated here. In turn, the amounts of extracted compounds containing sulfur (e.g., methanethiol, CS2) or nitrogen (e.g., pyrrole, methylpyrazine) were inversely proportional to extraction time, suggesting competition and perhaps displacement of these molecules from the HLB sorbent with analytes containing more electronegative oxygen in their molecules. Due to practical reasons, extraction times longer than 90 min were not studied.

### 2.2. VOCs Released from A549 Cancer Cells

Four Petri dishes were seeded with 250,000 A549 cells and cultivated over 4 days. After this time, viable cell count was checked with the Trypan blue dye exclusion method using an automatic cell counter (in triplicate for each biological replicate). The calculated cell loads were within the range of 1.83 × 10^6^–2.16 × 10^6^ cells/mL, while relative standard deviations ranged from 3.6% to 5.4%.

For the untargeted analysis of volatile metabolites produced by A549 cancer cells, the previously optimized protocol was used with extraction on HLB meshes in “internal-headspace” sampling mode at elevated temperature of 37 °C for over 90min.

**Figure 5:**
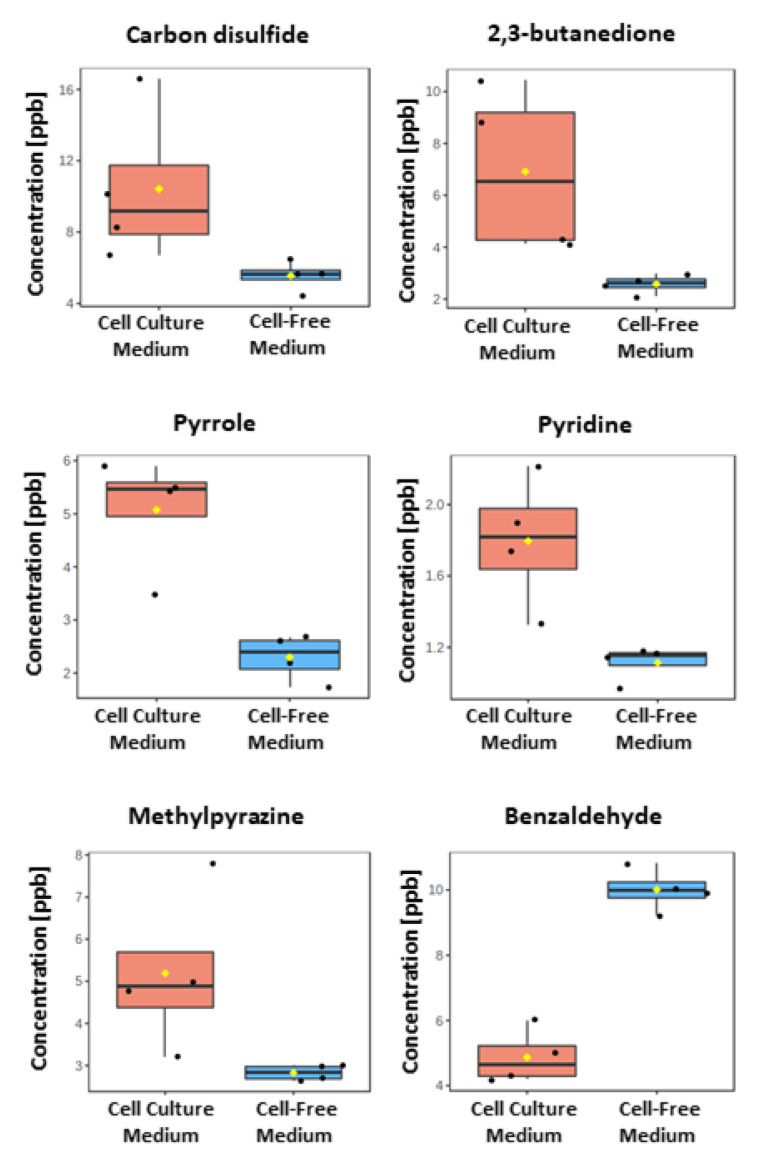
VOCs detected at significantly higher (respectively, lower) levels in the headspace of A549 cell culture medium (red boxes) compared to the reference sample of cell-free RPMI-1640 medium control (blue boxes). Boxes represent the 25th, 50th and 75th percentile, while whiskers refer to the 5th and 95th percentile. Mean values are indicated with a yellow diamond.

Out of the 40 VOCs unambiguously identified by confirmation of retention time of standards, in addition to the NIST Mass Spectra Library Search, six metabolites were observed at statistically different levels in the headspace of the cell culture medium compared to the cell-free medium controls at levels above their respective Quantitation Limit. Carbon disulfide, 2,3-butanedione, pyrrole, pyridine and 2-methylpyrazine were found at higher levels, whereas benzaldehyde was the only VOC observed at a decreased level in cell culture medium (Figure 5). The concentrations of the mentioned VOCs measured in both sample types, along with calibration parameters, are given in the Table 1. Our findings are consistent with the literature (Table 2), as the diminishing profile of benzaldehyde was previously reported in studies with 2D and 3D cultures of the A549 cell line [17]. Moreover, this compound exhibited decreasing profile also in other cell lines, such as fHB, HUVEC, HDF, NCM460, HepG2, RGP, VGP, Mm, SW1116, HeLa, SW480, compared to the respective control medium samples thoroughly reviewed in [20]. Uptake of aldehydes by cancer cells may be related to the increased activity of aldehyde dehydrogenase, which, amongst other biological functions, oxidizes the cytotoxic aldehydes into carboxylic acids. Intriguingly, Marcato et al. [21] indicated that only a certain number out of possible 19 isoforms of aldehyde dehydrogenase are active in particular tumor types. In turn, alcohols and ketones may originate from oxidation of alkanes by Cytochrome P450 oxidase enzymes, the activation of which in transformed cell lines was discussed by Watanabe [22]. While the origin of straight chained alkanes is related to the free radical peroxidation of polyunsaturated fatty acids (PUFA) [23], there are justified doubts that this pathway can lead to the production of methylated hydrocarbons [24].

Two out of six mentioned compounds were found to be significant in other in vitro studies focused on VOCs analysis in A549 cells [10,15,16,17,18], and four compounds were significant in ex vivo studies with cancerous lung tissues [8,19]. On the other hand, carbon disulfide and methylpyrazine were not reported for A549 cells nor for tumor tissues. Some compounds have consistent profiles amongst studies, such as decreasing levels of benzaldehyde, while other compounds do not exhibit such consistency. The reason for the observed divergence is currently unknown, but it may be related to different growth conditions, *inter alia*, adhesive cultures on the Petri dish in the RPMI medium vs. bottled 3D suspension in the DMEM medium, or different availability of oxygen, which influences the activity of enzymes and free radical oxidation. Relatively small number of analytes with statistical significance may be related to some extent to the data acquisition mode, where a single-quadrupole mass spectrometer operating in full scan mode was used to cover the widest range of *m*/*z* for appropriate untargeted analysis and identification of unknown analytes, instead of the selected ion monitoring (SIM) mode which drastically improves the sensitivity of MS analyses but focuses only on chosen *m*/*z* values.

Intriguingly, similarities between the results for the A549 cells and ex vivo studies on VOC emissions from surgically excised tumor tissue were observed, whereby pyridine was significantly produced from both these cancerous sample types compared to their respective nontransformed reference controls. The interdisciplinary convergence in VOCs release from tumor tissues and A549 cells was observed, also, for 2,3-butanedione and pyrrole; however, the increase in their levels from tumorous lung tissue did not reach statistical significance compared to reference healthy lung tissue in another study [19].

### 2.3. Study Limitations

In vitro models are very useful for protocol and methodology development as they offer strictly controlled and reproducible conditions while remaining cost effective and ethical, but they also have considerable limitations. In this regard, only a certain (relatively small) number of adherent cells could be studied at once due to the limited growth surface and capacity of the liquid medium in the Petri dish. The study was also limited by a small number of replicates and application of a sole A549 cell line. However, this work did not aim at the differentiation of cancer cells by means of detected VOCs but was intended to prove the potential application of a novel TFME technique to in vitro model investigation of volatile metabolites. Extensive research with different cell lines and more replicates is planned in the near future utilizing the sampling protocol optimized here.

## 3. Materials and Methods

### 3.1. Thin-Film Microextraction Devices (TFME)

TFME meshes were manufactured according to [25] and were kindly provided by Prof Pawliszyn (University of Waterloo, Waterloo, ON, Canada). Different chemistries of extraction phase were assessed: (1) pure PDMS, (2) hydrophilic-lipophilic balanced (HLB), (3) divinylbenzene (DVB), (4) Carboxen (CAR). All provided membranes had an initial dimension of 20 × 4.65 mm. However due to the diameter of the glass thermal desorption tubes, complementary with our autosampler sheets, they were cut into in half-widths (Figure 1). Always, before each sampling, membranes were preconditioned by placing them in 250 °C (pure PDMS, DVB, HLB) or 275 °C (CAR), for 30 min with a continuous flow of nitrogen 6.0 purified on a Carrier Gas Purifier (Agilent, Santa Clara, CA, USA) using a TD Clean-Cube conditioning unit (Scientific Instruments Manufacturer GmbH, Oberhausem, Germany).

### 3.2. Thermal Desorption Gas Chromatography Mass Spectrometry (TD-GC-MS)

The TFME sheets with adsorbed samples were placed inside a 3.5″ long and 1/4″ O.D. glass tube (Supelco, Bellefonte, PA, USA) for thermal desorption in a TD-30R autosampler (Shimadzu, Kyoto, Japan). Meshes composed of DVB, HLB and PDMS were thermally desorbed at 250 °C, with Carboxen meshes at 275 °C, for 10 min. As a carrier gas for thermal desorption, Helium 6.0 purified on an HP-2 Heated Helium Purifier (ViCi Valco Instruments, Houston, TX, USA) and a double set of Carrier Gas Purifiers (Agilent, Santa Clara, CA, USA) was used with a flow of 60 mL/min. The desorbed gases were focused on a cold trap at −20 °C filled with Carboxen. Injection into a Nexis 2030 Gas Chromatograph (Shimadzu, Kyoto, Japan) took place at 350 °C over 2 min in split-less mode with a carrier gas flow of 0.82 mL/min (linear velocity 33.0 cm/s). Chromatographic separation was done on an Rt-Q-Bond capillary column 30 m × 0.25 mm × 8 µm (Restek, Bellefonte, PA, USA) using the following temperature program: initial 60 °C held for 2min, then ramped 8 °C/min to 110 °C @1 min, ramp 3 °C/min to 120 °C @7 min, ramp 3 °C/min to 155 °C @7 min, ramp 3 °C/min to 225 °C @4 min, ramp 10 °C/min to 300 °C @7 min. Data acquisition was done with a QP-2020 Mass Spectrometer (Shimadzu, Kyoto, Japan), working in SCAN mode (within 33-235 *m*/*z* range).

### 3.3. Cell Cultures

For extraction protocol optimization (see Section 3.4) the same volumes of RPMI-1640 medium collected after cultivation of human adenocarcinoma A549 (Sigma-Aldrich, Taufkirchen, Germany), human breast cancer MDA-MB-231 (Sigma-Aldrich, Taufkirchen, Germany), human hepatoma Hep G2 (Sigma-Aldrich, Taufkirchen, Germany), human glioblastoma A-172 (CLS Cell Lines Services GmbH, Eppelheim, Germany) and mouse melanoma B16F10 (kindly provided by Dr Bajek, Nicolaus Copernicus University in Toruń, Poland) cell lines (at 100% confluence) were pooled. All cell lines were cultivated at 37 °C and 5% CO_2_ with RPMI-1640, L-glutamine and sodium bicarbonate (Sigma-Aldrich, Taufkirchen, German), 10% Fetal Bovine Serum (FBS, Biowest, Nuaillé, France), as well as antibiotics and antimycotic solution (Sigma-Aldrich, Taufkirchen, Germany). For the A549 biomarker discovery experiment, cells underwent a standard passaging procedure and then cell suspensions (Invitrogen by Thermo Fisher Scientific Inc., Waltham, MA, USA) were counted with 0.4% of trypan blue (dilution factor = 2) using a cell counter (Countess^®^ II FL, Invitrogen by Thermo Fisher Scientific Inc., Waltham, MA, USA) and seeded at a density of 250,000 per Petri dish, (100 mm in diameter). The medium volume was 10 mL. A reference sample was created by adding 10mL of complete medium (i.e., including 10% FBS) into the Petri dish. After 4 days of incubation, an aliquot of 5 mL of cell culture medium from the cell lines, and the same volume of reference cell-free medium samples, were centrifuged briefly (350× *g*, 1 min) to remove cell residue and transferred into a 20 mL glass vial sealed with a gas-tight cap. Simultaneously, cells seeded and cultures as described above underwent a standard passage procedure. Then, cells in suspension were counted as described above to determine the total number of cells after 4 days of culture. For each sample type, four independent replicates were used (*n* = 4).

### 3.4. Sampling Optimization

#### 3.4.1. Selection of Sorbent

The pooled cell culture medium was used for extraction on pure PDMS, DVB, HLB and CAR TFME sheets to ensure a relevant sample matrix with the richest content of diverse VOCs. For this purpose, 5 mL sample was transferred to a 20 mL glass vial and sampling was conducted for 30 min according to protocol “B” described in Section 3.4.2. For each sample type, four independent replicates were used (*n* = 4).

#### 3.4.2. Selection of Sampling Protocol

TFME extractions were carried out according to two different protocols using the same pooled RPMI 1640 culture medium. For each sampling mode, three independent replicates were used (*n* = 3).

**Figure 6:**
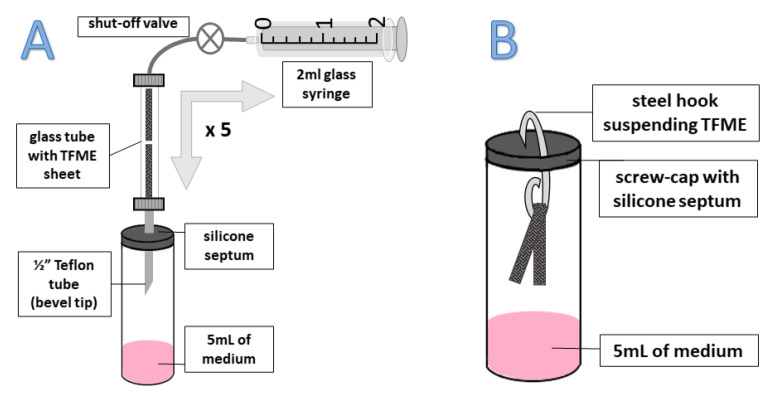
Modes of headspace sampling: (**A**) External headspace where TFME mesh was placed inside a sorption glass tube exposed to the sample via a 1/4″ Teflon tube (flushed five times bidirectionaly prior to extraction), (**B**) Internal headspace, where TFME mesh was suspended on a steel hook inside the tightly closed 20 mL glass vial containing 5 mL of liquid sample.

Protocol A, referred as external headspace (EX-HS). Once the 5 mL of pooled medium was placed in 20 mL gas tight vial, the initial headspace was purged with nitrogen 6.0 (additionally purified on Agilent Carrier Gas Purifiers) for a short period to remove exogenous contaminants and left for 30 min at room temperature (20 °C) to reach a new equilibrium in the headspace. A 1/4″ Teflon tube sharpened to a bevel tip was then introduced to the vial through a silicon septum in a screw-cap and connected by means of a Swagelok 1/4″ straight unit to the sorption glass tube containing TFME sheets, fixed by glass wool. Five cycles of bidirectional transfer of 2 mL of headspace gas through a glass tube with TFME sheets were done with a glass syringe (Socorex Isba SA, Ecublens, Switzerland) to equally distribute VOCs in the system. Afterwards, a shut-off valve placed behind tube with TFME sheets was closed for a 30 min long extraction (Figure 6A).

Protocol B, referred to as internal headspace (IN-HS). TFME sheets were suspended on a steel hook under the silicon septum in a screw-cap inside the 20 mL glass vial containing 5 mL of pooled medium (Figure 6B). Extraction took place at ambient temperature (20 °C) over 30 min. Afterwards, TFME sheets were detached from the hook and tightly closed in a sorption glass tube.

#### 3.4.3. Extraction Temperature Optimization

The headspace from 5 mL of pooled medium was collected on HLB mesh according to the protocol B (IN-HS) described in Section 3.4.2. The only variable was the temperature of the 30 min long extraction, whereby 20 °C “cold” sampling and 37 °C “heated” sampling was compared. None of the other activities before and after extraction were changed. For each sampling type, three independent replicates were analyzed (*n* = 3).

#### 3.4.4. Extraction Time Optimization

Each sampling was performed on HLB meshes according to t protocol B (IN-HS) at an elevated temperature of 37 °C. Extraction times of 10, 20, 35, 60 and 90 min were compared. TFME with adsorbed samples were detached from the hook and stored in tightly closed glass tubes until GC-MS analysis. For each extraction time, three independent replicates were analyzed (*n* = 3).

### 3.5. Calibration of Target VOCs

Compounds for which statistically significant differences were observed between cell culture medium and cell-free medium were calibrated by addition of pure standards into the RPMI1640 medium supplemented with 10% FBS in an amount appropriate to reach the desired final concentration. The headspace from the samples was then extracted with TFME according to the optimized sampling protocol using HLB meshes. To ensure the highest quality of our data, Detection Limits and Quantitation Limits were calculated, not from the S/N ratio for repeated blank measurements, but were determined from the calibration curve of a respective standard according to the “Validation of Analytical Procedure” guidelines after the European Medicines Agency [26] using following equations:

$$DL=\frac{3.3\cdot \sigma }{S}$$

and

$$QL=\frac{10\cdot \sigma }{S}$$

where:

*σ*—standard deviation of the Y-intercept of the regression line
*S*—slope of the regression line

### 3.6. Data Processing and Statistical Analysis

Detected compounds were identified first by matching the acquired spectrum with the NIST 2017 (Gaithersburg, MD, USA) Mass Spectra Library and additionally confirmed by the retention time of a reference substance. All substances used for identification of detected metabolites were purchased from Alchem (Alchem, Toruń, Poland). Chromatographic peaks were initially integrated using a customized method in GCMS Postrun Analysis software (Shimadzu, Kyoto, Japan) and, if necessary, manually corrected by an experienced analyst. A received compound table was produced in Metaboanalyst 5.0 online software [27], where peak areas were log-transformed. The statistical significance between VOC levels in A549 cells and reference the RPMI1640 medium was calculated in Statistica 13.3 PL software (StatSoft, Inc., Tulsa, OK, USA) using the U Mann-Whitney test, which is a nonparametric test to compare samples from two groups of independent observations, where *p*-values < 0.05 were considered to be significant. This test was chosen due to its stability to outliers and no requirement for the groups to be normally distributed. To determine LOD/LOQ and summarize the data, results of calibrations and optimization measurements were plotted using Microsoft Excel, while the results of in vitro experiments with A549 cells were plotted using Metaboanalyst 5.0 online software [27].

## 4. Conclusions

For the first time, the TFME technique was successfully used in the investigation of volatile metabolites originating from cancer cells. Selection of optimal sampling parameters allowed analysis of 68 volatile metabolites in a wide range of volatilities and polarities. Identification of 40 VOCs was confirmed by retention time of standards in addition to a NIST mass spectra library search. Out of these, five volatile metabolites were significantly released by A549 cancer cells, while benzaldehyde was the sole VOC that decreased. Compounds previously unreported for A549 cells were observed, including carbon disulfide and methylpyrazine. Similarity in pyridine profiles in an ex vivo study with lung tumor tissue, and in vitro A549 cell lines indicates that this metabolite can be related to oncogenic transformation. This study provides a good basis for further application of TFME protocols to in vitro experiments with cell cultures focused on identification of volatile cancer-related metabolites potentially useful for early diagnosis of lung cancer.

## Figures and Tables

**Table 1:** Concentration [ppb] of target volatiles measured in the headspace samples of cell culture medium and cell-free reference medium along with calibration parameters. Concentrations higher than LOD but lower than LOQ are labeled with an asterisk.

Compound Name	CAS	R^2^	LOD [ppb]	LOQ [ppb]	Cell Culture Medium		Cell-Free Medium	
					Mean Conc. [ppb]	Standard Deviation [ppb]	Mean Conc. [ppb]	Standard Deviation [ppb]
Carbon Disulfide	75-15-0	0.9957	1.360	4.121	10.423	4.343	5.552	0.851
2,3-Butanedione	431-03-8	0.9880	2.280	6.909	6.918	3.184	2.581 *	0.370
Pyrrole	109-97-7	0.9986	0.782	2.370	5.073	1.087	2.297	0.435
Pyridine	110-86-1	0.9986	0.763	2.313	1.796 *	0.369	1.114 *	0.097
Methylpyrazine	109-08-0	0.9948	1.496	4.533	5.191	1.916	2.827 *	0.185
Benzaldehyde	100-52-7	0.9987	0.747	2.264	4.872	0.823	10.011	0.654

**Table 2:** Compounds identified as statistically important for A549 conditioned vs. control medium with respective relation to literature data.

Compound Name	CAS	Best TFME Type	Best Extraction Time [min]	Literature Finding	Literature Sample Prep.	Reference
2,3-Butanedione	431-03-8	HLB	10	ex vivo human lung tissue	Tenax-CAR TD tubes	[19]
Benzaldehyde	100-52-7	HLB	90	A549 cells	CAR-DVB SPME fiber	[10]
				ex vivo human lung tissue	CAR-DVB SPME fiber	[8]
				A549 cells	Graphite and C18 silica monolith monotrap	[17]
				ex vivo human lung tissue	Tenax-CAR TD tubes	[19]
Carbon Disulfide	75-15-0	PDMS	10			
Methylpyrazine	109-08-0	HLB	10			
Pyridine	110-86-1	CAR	35	ex vivo human lung tissue	Tenax-CAR TD tubes	[18]
Pyrrole	109-97-7	CAR	10	A549 cells	CAR SPME fiber	[9]
				A549 cells	Tenax-CAR TD tubes	[18]
				ex vivo human lung tissue	Tenax-CAR TD tubes	[19]

## Data Availability

The data presented in this study are available in article.
